# Fermented banana feed and nanoparticles: a new eco-friendly, cost-effective potential green approach for poultry industry

**DOI:** 10.1016/j.psj.2025.105171

**Published:** 2025-04-17

**Authors:** Muhammad Saeed, Faiz‐ul Hassan, Hanan Al-Khalaifah, Rafiqul Islam, Asghar Ali Kamboh, Guiqin Liu

**Affiliations:** a College of Agriculture and Biology, Liaocheng University, Shandong Engineering Technology Research Center for Efficient Breeding and Ecological Feeding of Black Donkey, Shandong Donkey Industry Technology Collaborative Innovation Center, Liaocheng, 252000, PR China; b Faculty of Animal Production & Technology, Cholistan University of Veterinary and Animal Sciences, Bahawalpur, 63100, Pakistan; c Environment and Life Sciences Research Center- Kuwait Institute for Scientific Research, Safat, 13109, Kuwait; d Department of Poultry Science, College of Veterinary Science, Assam Agricultural University, Guwahati, 785013, India; e Department of Veterinary Microbiology, Faculty of Animal Husbandry and Veterinary Science Sindh Agriculture University, 70060 Tandojam, Pakistan

**Keywords:** Banana feed, Prebiotics effects, Nanoparticles, Natural growth promoters, Poultry

## Abstract

The quest for sustainable, alternative, and cost-effective biofeed resources has been driven by the increasing costs and environmental concerns linked to conventional poultry feed. The banana plant (*Musa* spp.), traditionally valued for its fruit, is gaining recognition as a versatile and sustainable resource for the livestock and poultry industry. Rich in essential nutrients, fibers, and bioactive compounds, banana by-products enhance animal health, improve digestion, and reduce feed costs. Studies reveal that banana plant have potential as natural growth promoters, prebiotics, and antioxidants, contributing to improved feed efficiency and resilience against diseases. The peel of a banana is a good source of vitamins, crude protein (6–9 %), starch (3 %), total nutritional fiber (43.2–49.7 %), and crude fat (3.8–11 %), making it good source of nutrition for animals and birds. In addition, banana’s peels contain flavonoids, tannins, phlobatannins, alkaloids, glycosides, and terpenoids. These compounds have antibacterial, growth promoter, antioxidant, stress reducer, anti-cholesterol, antihypertensive, immunostimulants, and anti-inflammatory properties. The banana plant, which is often regarded as agricultural waste, is rich in carbohydrates, fiber, and essential minerals, making it a valuable feed component. Results from experimental studies showed improved feed conversion efficiency, growth performance, and gut health in poultry fed with fermented banana plant-based diets. Fermentation improves the nutritional quality of banana plant biomass by increasing digestibility, reducing anti-nutritional factors, and enriching it with probiotics and bioactive compounds. The fermented banana had a substantial influence on weight gain and feed consumption in chickens. Banana meal may be used into broiler chicken diets at a maximum of 10 % without negatively impacting productivity. Silver nanoparticles (nano-Ag) produced by the banana plant can be used as an alternate growth-promoting supplement for poultry production. This approach offers environmental advantages by minimizing agricultural waste and encouraging more sustainable poultry production practices. Overall, the available studies highlight the considerable promise of fermented banana plants as a sustainable and environmentally friendly option in poultry feeding, tackling both economic and ecological issues faced by the poultry sector.

## Introduction

Feed expenses represent the most significant recurring cost in poultry production, so substantially influencing the industry's profitability and the affordability of poultry products for customers. The rising demand for cereals in the human food industry and the diversion of maize for biofuel production are the primary reasons contributing to the significant increase in poultry feed prices in recent years. Rising feed component costs significantly adversely affect animal husbandry output and consumption, especially in poor nations (Beken and Şahin, 2011; Tsiouni et al., 2021; Andika et al., 2025). Competition for resources, including land for industrialization and grains for human consumption, escalates production costs and limits commercial operations for farmers who dependent on traditional cereals and meal sources in animal diets (Vasta, et al., 2008). Despite rising demand for livestock/poultry products in most developing countries, many are experiencing a feed deficit and unable to source enough animal feed to raise healthy livestock and poultry. Because the climate is not ideal right now, it is important to use non-traditional feed sources to create a sustainable livestock/poultry diet plan (Gertenbach and Dugmore, 2004; Katongole, et al., 2011; Heng, et al., 2022; Saeed, et al., 2023, 2025; Kadhim, et al., 2025; Koni et al., 2025; Shi et al., 2025).

To overcome these obstacles and boost animal performance, suitable technical advancements should be made to increase its feeding value and storage quality of alternate feed sources (Wu et al., 2024; Yan et al., 2024). It’s important to use non-traditional feed sources, like the banana, also known as *Musa paradisiaca* and belonging to the family Musaceae, is a widely cultivated kind of fruit. It is perhaps the oldest crop that has ever been cultivated anywhere in the world and is one of the tallest herbaceous plants that has a pseudo stem (Kumar et al., 2012). With a share of 65–70 % of the overall production cost, feed is the most costly ingredient in the chicken production industry (Abel et al., 2014; Maina et al., 2012). Banana peel serves as an alternate feedstuff for animal nutrition, obtainable from local banana market vendors and the banana processing sector (Tewe, 1983). Banana peel provides a substantial source of starch (3 %), crude protein (6–9 %), crude fat (3.8–11 %), total dietary fiber (43.2–49.7 %), and vitamins, making it suitable as animal and poultry feed (Debabandya et al., 2010; Zhang et al., 2005). Conversely, ruminants can and should benefit greatly from a diet that includes leftover banana (*Musa acuminata*) (Dubale, 2017). Bananas, extensively cultivated in Africa, are abundant in potassium and calcium while being low in sodium content, making them highly recommended for individuals with hypertension (Adisa and Okey, 1987; Chandler, 1995; Anhwange, 2008). Dried banana peels (DBP) may be used up to 18 % as alternate feedstuffs for laying hens without adversely affecting performance and egg quality features (Shumye et al., 2022). To reduce anti-nutritional factors in banana pericarp (peel) for poultry use, various treatment methods were used, such as adding ash and covering for 3-5 days, sun drying for 4-5 days, oven drying for 2 h at 100°C, and using alkali and sodium hydroxide (Atapattu and Senevirathne, 2013).

A number of researchers have documented the utilization of banana peel meal in broiler hens, while others have described the incorporation of waste banana fruit in the diet of pigs and revealed a positive effects on production (Tewe, 1983; Forster et al., 2003; Atapattu and Senevirathne, 2013; Duwa et al., 2014). When compared to the mesocarp (pulp), the peel of the banana has a higher concentration of crude fiber and ash, but a lower concentration of crude protein. As a result, it is an excellent alternative feedstuff for animal feeding (Oluyemi and Roberts, 1981). There have been a number of studies that have reported the utilization of banana peels in chicken feeds as a means of partially substituting maize as a source of energy (Abel, et al., 2015; Blandon, et al., 2015; Duwa et al., 2014). Banana peel comprises 10 % crude protein and 2932 kcal/kg of metabolizable energy (ME). Currently, there is no specific recommendation is available in the literature suggestion for the optimal inclusion level of banana peel in chicken feeds is available in the literature (Blandon et al., 2015). Researchers have observed that *Macrobrachium rosenbergii* exhibited enhanced growth, immunity, and resistance to *L. garvieae* when it was provided with diets that contained banana peel extract (BPE) at doses of 0 g kg−1, 1 g kg−1, and 3 g kg−1. They concluded that this dosage of BPE promoted growth, reduced hyperthermal stress, and improved resistance (Rattanavichai and Cheng, 2015). When rye-grass hay was replaced with banana byproducts, scientists examined the effects on feed consumption, ADG, and FCR in Pelibuey lambs, a hair sheep breed. This study suggests that supplementation of banana byproducts instead of usual fodder serve as an effective alternate for feeding subtropical Pelibuey lambs (Barbera et al., 2018). Another study using banana peels powder (adding 4 % of concentrated diet) reported notable increases in body weights and dry matter intake in all treatment groups in Iraqi goat's kids (Al-Absawi et al., 2020).

Banana peel contains about 0.9 %, crude protein, 1.7 %, 59.0 % crude carbs, and 31.70 % crude fiber. If it is prepared properly, it has the potential to be an excellent and inexpensive source of carbohydrates and minerals for animals (Anhwange, 2008).

Published literature suggested that banana plant and residual products become more nutritious through fermentation. So, it could be a potential an alternative feed resource, that can be used to meet the poultry needs of the increasing population. The current review paper concludes that fermented banana plants and their therapeutic potential could serve as a viable alternative poultry feed resource. The goal of this study is to lay the groundwork for more research into how fermented banana plant and its bioactive compounds can be effectively used in raise poultry farming. Chemical composition of banana fruit is presented in (Table 1).

**Table 1:** Chemical composition of banana fruit (per 100 g) *.

**Parameter**	**Content (g)**
Protein	0.9–4.9
Crude fiber	1.6–2.9
Energy	371 kJ (89 kcal)
Lipids	0.3–2.9
Sugar	23.9–43.8
Ash	0.9–2.22
**Vitamins**	**Mg**
Vitamin C	8.7 (10 %)
Pyridoxine	0.4 (31 %)
Pantothenic acid	0.334 (7 %
Choline	9.8 (2 %)
Minerals	**Mg**
Potassium	358 (8 %)
Magnesium	27 (8 %)
Zinc	0.15 (2 %)
Phosphorus	22 (3 %)
Sodium	1 (0 %)

⁎ Adopted from (Dotto, et al., 2019; Kookal and Thimmaiah, 2018).

## Methodology

This review analyzes publications addressing fermented banana feed as a novel, eco-friendly, and cost-effective resource in the poultry business, along with studies emphasizing its potential application in silver nanotechnology. The information for this review was obtained from esteemed platforms like SpringerLink, Scopus, Web of Science, PubMed, Google Scholar, and Science Direct**.** Several key terms were employed in the search, including fermentation, banana feed, its bioactive components, growth performance advantages, and silver nanoparticles. The criteria for article selection were stringent, concentrating exclusively on studies published in esteemed, peer-reviewed English-language publications from 2012 onward, with the exception of two older studies from 2006. Conference summaries, books, and sections thereof were excluded from this review.

### Banana plant

**Fig. 1:**
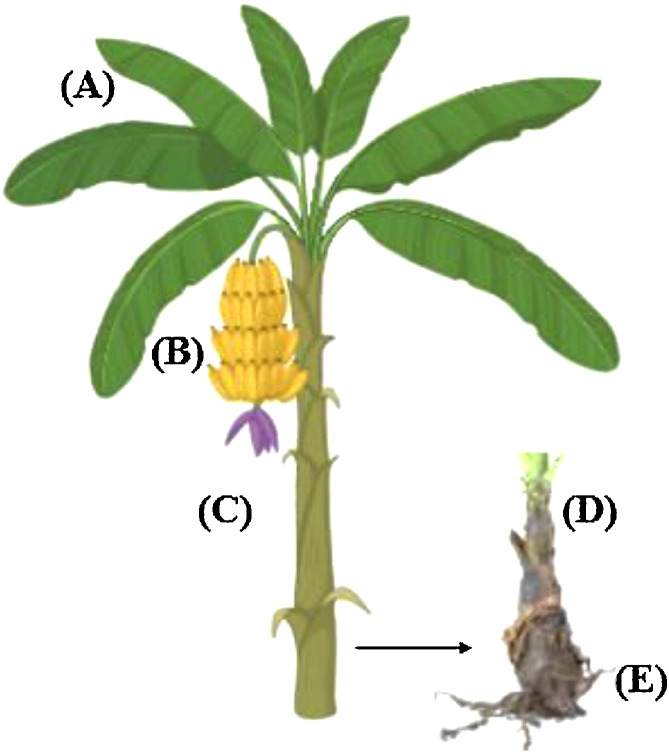
Banana plant parts. (a) Leaves, (b) Fruits, (c) Flower, (d) Pseudo-stem, (e) Root.

The banana is an ancient plant with a cultivation history dating back around 10,000 years. The banana pseudo-stem (BPS) can attain a height of 6–7.6 meters, supporting fruits, flowers, and leaves (Fig. 1). Banana leaves are substantial, measuring up to 2.7 × 0.6 meters, flexible, and impermeable to water. They are appropriate for packaging and presenting food because of their extensive waxy surface (Ahmadi, et al., 2019). They are also abundant in fibers, tannins, polyphenols, and flavonoids (Al-Mqbali and Hossain, 2019). The leaves are conventionally employed to address several skin ailments, including eczema, cuts, inflammation, rashes, dandruff, and sunburn, owing to their cooling properties (Kora, 2019). The pseudo-stem, a significant part of plant biomass, is often left on plantation land or burned and wasted. Drinking fresh stem juice can cleanse the body, prevent kidney stones, and treat epilepsy, dysentery, and diarrhea (Qamar and Shaikh, 2018). Bananas contain high concentrations of bioactive chemicals, such as glycosides and acids like malic acid and oxalic acid, in addition to their high proportion of sugar derivatives, polyunsaturated fatty acids, sterols, minerals (e.g., potassium), and various vitamins (e.g., provitamin A, B1, B2, C) (Mathew and Negi, 2017). Type II resistant starch and non-starch polysaccharides, such as dietary fiber, are abundant in the green banana and its product, whole green banana flour (WGBF) (Tepal, 2016). WGBF has been integrated into a variety of culinary products, including bakery products, baby foods, pastries, dry noodles, and pasta (Türker, et al., 2016; Garcia-Valle, et al., 2019). The edible parthenocarpic (seedless) banana pulp changes into various skin colors, such as yellow, purple, orange, and red, as it ripens. Red bananas have significant levels of lutein and lycopene, while yellow and orange-fleshed bananas are known to be high in trans-β-carotene. Both prevent age-related macular degeneration and offer men protection against prostate cancer (Sidhu and Zafar, 2018). Especially, plantains and green banana pulp (GBP) can be cooked into fried bananas, banana chips, and banana butter (Ferreira and Freitas, 2019).

### Application of Banana Plant in Terms of Sustainable Poultry Farming

Since the 21st century, poultry production has aligned with sustainable practices and termed as ‘sustainable poultry production’. It refers to a system of raising poultry while ensuring the health of the environment, animal welfare, and economic viability (Castellini et al., 2006; Leinonen et al., 2016; Bist et al., 2024). The use of banana plants in poultry farming meets these criteria thus ensuring sustainability in poultry farming. Waste reduction and eco-friendliness can be achieved by using banana peels, leaves, and pseudostems as poultry feed. Integrating these parts of banana plant aligns with sustainable agricultural practices in multiple ways, but mainly through the harmonious integration of the following 3 dimensions (Vaarst et al., 2015):

**Waste Reduction:** Effective waste management and resource efficiency are important pillars of sustainable poultry faming. Proper handling and use of agricultural waste can help reduce environmental pollution, conserve resources, and create additional benefits for the community (Rauw et al., 2023). Through the utilization of banana byproducts, agricultural waste is reduced, which contributes to the development of a circular economy in which resources are reused in an effective manner.

**Environmental Impact:** To improve the environmental sustainability of poultry production, the exploration of alternative feed resources is critical that have minimal or no environmental foot prints (Leinonen et al., 2014). By decreasing reliance on conventional feed ingredients like maize and soybean, it is possible to reduce the environmental footprint associated with their cultivation, which includes the destruction of forests and the consumption of a significant amount of water (Van der Werf et al., 2005).

**Economic Viability**: In the search for sustainable and affordable alternatives to conventional feed resources, locally available agricultural by-products present a promising opportunity (Cui et al., 2021). Among these, banana plants stand out due to their abundance and accessibility in many tropical and subtropical regions. Considering that banana plants are frequently found in the immediate vicinity and are very inexpensive, they offer a cost-effective alternative to feed that has the potential to increase the profitability of poultry farming operations.

### Banana plant as animal feed and fodder

Nowadays, there is interest in producing industrial flour from green bananas due to their nutritional value, particularly the large amount of resistant starch (40.9–58.5 %) and dietary fiber (6.0 %–15.5 %) (Tribess et al., 2009; Oliveira et al., 2014). It possesses a high starch content and is extensively utilized in animal feed. Despite being a valuable nutritional component, the underutilization in animal diets represents a significant nutritional deficiency due to the presence of extractable bioactive chemicals, which can serve as value-added materials (Martinez et al., 2009; Safwat et al., 2014).

Banana peel is a significant source of dietary fiber and pectin. The banana by-products have been converted into animal feed. (Wachirasiri et al., 2009; Álvarez et al., 2015; Khamsucharit et al., 2018). In the existing body of research, banana peels have been shown to be utilized in the production of feed for ruminants (Rigueira et al., 2021). Parts of the banana plant, including the leaves, juvenile plants, discarded fruit, and stems, can serve as fodder for ruminants, especially cattle, sheep, goats, poultry and buffalo (Ffoulkes and Preston, 1978a). To serve as a partial substitute for sorghum silage, banana byproducts may be employed to nourish ¾ Holstein × ¼ Zebu heifers. This is because banana wastes do not affect the animals' natural weight gain or body development (Rigueira et al., 2021). The nutritional worth of banana by-products, encompassing leaves, pseudostems, and raceme stems, was examined for goats utilizing rumen degradability and in vitro digestibility methods. However, it is important to note that rations consisting solely of banana leaves should be able to fulfill more than 85 % of the requirement for maintenance energy (Pieltain et al., 1999). It is recommended to use solely the leaves is recommended in order to achieve the best possible results in terms of anthelmintic and nutritional characteristics (Marie-Magdeleine et al., 2010). Supplementing Nile tilapia with banana flower powder (BFP) been shown to stimulate growth in these fish (Phinyo et al., 2024). Vietnamese fish farmers use fresh maize and banana leaves as feed supplements, which may have phyto-prophylactic benefits and improve growth performance. Leaf supplementation also protected fish from intramuscular A. *hydrophila* injection (Mayrhofer et al., 2017). The fermentable byproducts of ripe bananas, which include banana peels and pulp remnants from the wine fermentation process represent a major waste source with possible uses. Current research, supports the inclusion of protein, polysaccharides, and dietary fibers in banana byproducts qualifying them as potential ingredients for animal feed production (Padam et al., 2014). It proved that giving banana peel to cattle increased their live body weight and FCR (Hernan Botero et al., 2000).

### Banana plant as feed supplement

Potentially banana plant fibers in chick feed could serve as an alternative to antibiotics. Treatment groups comprised of T1 (basic diet without replacement), T2, T3, and T4 with fermented banana peel substitutions at 5, 10, and 15 % respectively. Dietary substitution with banana peel supplementation in native hens reduced total cholesterol, triglycerides, and HDL levels. The fermented banana peel can be utilized as an alternative nutrient supplement in native chicken feed, perhaps enhancing productivity in poultry (Nuriyasa et al., 2022). The incorporation of banana peel at a level of 20 percent in the diet of broilers experiencing heat stress can nevertheless yield favorable outcomes in final broiler weight, carcass percentage, and belly fat percentage within acceptable limits (Widjastuti and Hernawan, 2012). When compared to the control diet, the treatment using banana leaf produced a notable increase in yolk coloration (*p* < 0.05). This resulted in eggs that had a greater amount of orange pigmentation in their yolks. Regarding the examination of endoparasites, it was observed that there was a noteworthy impact (*p* < 0.05) associated with the proportion of infections that were found in the excreta (Silva et al., 2019). The banana peel can become a viable feed supplement. Proteins, carbs, fat, moisture, and ash are contained in banana peels, along with significant amounts of carotenoids (beta, alpha, and lutein) and phenolic chemicals. Oxidative stress-related diseases are strongly inhibited by phenolic compounds. Animal studies reveal banana peels promote immune response and antioxidant capability (Abasubong et al., 2024). It was determined that banana peel meal may be incorporated up to 10 % in broiler chicken diets without negatively impacting the birds' performance, hence enhancing the poultry production industry (Abel et al., 2015). Researchers explored banana flower powder (BAFLOP) to see if it might be utilized as a rumen buffering agent. They found that it raised the pH of the rumen, made nutrients easier to digest, and increased fermentation end-production. At 20–30 g/kg of DMI, it worked as a rumen buffering agent (Kang et al., 2014). In one study, adding banana flower powder was looked into, and it was suggested that BAFLOP pellets (60 g/kg of food substrate) could be used to change how rumen fermentation works in animals that are fed a high-concentrate diet (Kang et al., 2016). The trial included 200 male chicks aged two days. Four fifty-chick treatment groups were randomly assigned birds. Dietary supplements consisted in a basic diet (CON), one with 0.5 % banana as prebiotic (PRE), 0.2 % commercial probiotic Toyocerin® (PRO), or 0.7 % combination (SYN). The study found that bananas could be used as a feed additive to enhance growth performance from 1 to 21 days of age without affecting carcass or meat quality (Buranawit and Laenoi, 2015). In another study it was found that replacing dietary maize with modified banana tuber meal (M-BTM) improved growth and digestibility in growing-finisher colored-feathered hybrid ducks (Pekin x Khaki Campbell) (Sjofjan et al., 2021).

### Banana plant as meat quality enhancer

The birds fed fermented banana peels and pulps demonstrated enhanced slaughter performance and meat quality, especially in the Pu-10 group, relative to the control chickens (Li et al., 2024). Comparing the breast meat of hens fed fermented banana peels to meat samples taken from the control group, a higher lightness value was noted. This was observed in comparison to the hens that were fed the control group (Nuriyasa et al., 2022). Microbial fermentation of banana peels may improve taste and nutrition. The scientific literature shows that Lohmann broiler chicks fed fermented banana peels from day 22 to day 38 had better slaughter performance and meat quality than the control group (Sugiharto et al., 2020). One in another study, it was found that adding green banana biomass (GBB) and green banana flour to meat products revealed a feasible and favorable, improving chemical quality without sacrificing technological or sensory quality, and so reducing food waste in long run with the disposal of possible green banana by-products (Stragliotto et al., 2022). Egyptian banana peel extract has antibacterial activity against aerobic, *Enterobacteriaceae*, and S. *aureus* bacteria and can be used as a natural preservative for meat and meat products (Hafez and Eissawy, 2018). Because of its practical and technical uses, green banana biomass (GBB) is an intriguing component. The flavor of the chicken bologna was boosted in the formulation that used GBB to replace half of the additional fat(Auriema et al., 2021). Existing scientific literature provides evidence that fruit peels have beneficial impacts on the slaughter performance of chicken as well as the quality of the meat produced by poultry (Sugiharto, 2023; Wardani et al., 2023).

### Banana plant on egg production and quality

**Fig. 2:**
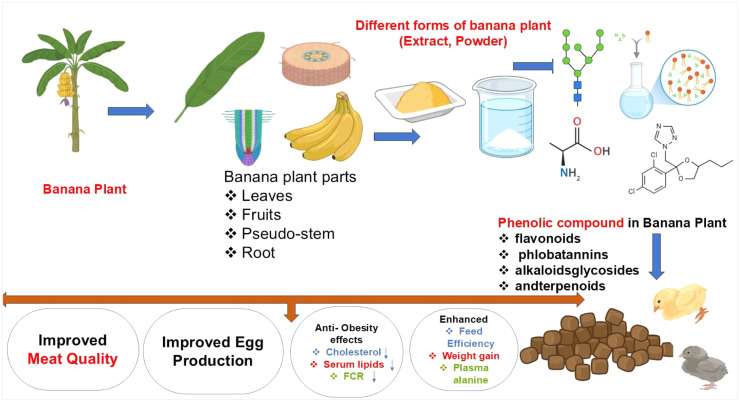
Promising beneficial effects of banana plant and its phenolic compound in poultry.

A dietary banana leaf content of 3 % resulted in a rise in egg weight and egg production, as well as an improvement in egg quality measures and a reduction in serum total cholesterol (Kanbur et al., 2023). Banana waste in dietary concentrations of 3 %, 6 %, and 9 %, with or without 1000 mg of multienzyme, enhanced egg production, particularly at the 3 % level, but the 9 % concentration diminished both egg weight and production output. All diets enhanced the yellowness of egg yolks and reduced their redness (Kanbur et al., 2023) found that the 9 % diet enhanced health and biochemical indicators more than a control. Understanding banana peel effects on laying hens' diets requires more research. There is a possibility that the substitution of ensiled banana leaves into the diet of laying hens will result in an increase in the amount of feed consumed as well as the cost of producing eggs. The eggs’s quality was unaffected by the ensiled banana leaves, though. Consequently, in order to enhance the quality of banana leaves, additional research is required (Yikhio et al., 2016). A study looked at how the Kepok Banana (*Musa Paradisiaca* L.) affected the immunoglobulin, vitamins, and cholesterol levels in eggs from laying hens. The results showed that the Kepok Banana had a good effect on the levels of lgY' and vitamins in eggs. It might also lower the amount of fat in eggs (Leke et al., 2022). Feeding laying hens banana stalks as part of a free-range rearing system in a bamboo forest (FBS-FRRS). When the eggs were raised in a battery cage (CON) or FBS-FRRS, the shell color score was much higher, as were the brightness and yellowness values of the boiled yolk (*P* < 0.05) (Nopparatmaitree et al., 2022). The Green Banana Flour coating is a good way to blend the egg shell and innards to lower the number of microbes that are present and keep the eggs' taste and quality inside (Oliveira et al., 2023). In one study, it was found that giving chickens 25 % banana peel powder mixed with corn-based feed helps them gain the most weight and lay the more eggs (Araya et al., 2021). Summarized different studies on favorable benefits of supplementation of banana plant on poultry production (Table 2). Promising effects of banana plant fed to poultry (Fig. 2).

**Table 2:** Summarized different studies on favorable benefits of supplementation of banana plant on poultry production.

Item	Effects	References
Banana flour	Banana flour improved laying hens' weight gain, body weight, and feed intake.	(Dumorné, 2018)
Banana leaves	Quail diets that contain 6 % banana leaves (BL) may improve the quality of the meat and the levels of HDL without having an effect on the development of the birds.	(Kanbur, et al., 2024)
Fermented banana leaves	In a study, broiler diets were supplemented with 5 %, 10 %, and 15 % fermented banana leaves using *Trichoderma viride*, revealing that 10 % fermented leaves, improved feed consumption, weight accumulation, feed conversion efficiency, and carcass yield of the hens.	(Mandey, Leke, Kaunang and Kowel, 2015)
Banana leaf powder	The use of 50 g/kg of banana leaf powder in broiler diets improved final body weight (FBW) and weight gain (WG), leading to improved feed conversion ratio (FCR).	(Oleforuh-Okoleh, et al., 2015)
Banana meal	Up to 15 % of corn can be replaced with banana peel meal without hurting the performance or blood components of broiler chickens. At the same time, feed costs per kg gain and N/kg feed costs go down.	(Duwa, Saleh, Lamido and Saidu, 2014)
Banana peels	Adding banana peels to the feed can help broilers handle heat stress better.	(Hernawan, 2014)
Banana peel	Adding banana peel to broiler feed up to a level of 3.0 % is cost-effective and helps the birds grow.	(Siyal, et al., 2016)

### Fermented banana plant and its residue as a nutritious biofeed

In animal production, feed costs account for 70 % of overall costs, with predicted increases over time (Mira et al., 2018; Rahman et al., 2020). Using banana crop leftovers as ruminant feed helps save feed costs for animal nutrition (Ffoulkes and Preston, 1978b). Due to the fact that banana leaves contain a large amount of moisture and a low amount of dry matter, they are not suitable for direct feeding to ruminants. It can be pre-treated by ensiled it by fermentation process with addition of chemicals and supplements to enhance the dry matter content and minimize the moisture content, so increasing the quality. Local banana crop residue allows local farmers to create their own silage instead of depending on imported feed (Álvarez et al., 2015; Jais and Rashid, 2017). The purpose of study was to investigate the nutritional composition, mineral content, and physical characteristics of banana leaves that had been fermented with molasses, effective microorganisms (EM), and urea. A substantial increase in crude protein content was observed (*P* < 0.05).Fermenting banana leaves with sugar, EM, and urea for 21 days enhances their nutritional value, making it a suitable pre-treatment option for animal feed (Mat et al., 2020).

**Fig. 3:**
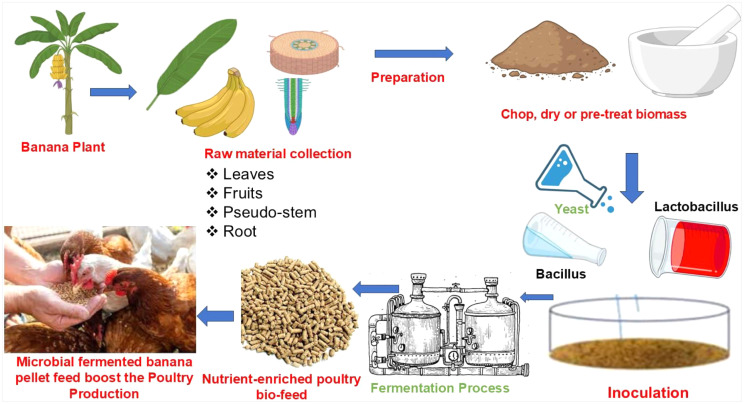
Flow diagram depicted the process of fermented banana feed and its effects in poultry.

There have been reports that various portions of the banana plant, such as the leaves, the peel, the stem, and so on, have been traditionally fed to ruminants and fish in many regions of the world. This may be owing to the high fiber content of the banana plant (Wachirasiri, Julakarangka and Wanlapa, 2009) and pectin (Khamsucharit et al., 2018) content. Various components of the banana plant may serve as an alternate energy source in broiler diets among agro-industrial by-products (Sugiharto et al., 2020). Also, banana peel has 2932 ME kcal/kg of crude protein and 10 % crude protein (Blandon et al., 2015). There have been reports that the peel of bananas contains a variety of bioactive components, including phenolic substances such flavonols, hydroxycinnamic acids, flavan-3-ols, and catecholamines (Zaini et al., 2022). These compounds containing phenolic compounds exhibit actions that are antioxidant, antibacterial, and antibiotic (Fidrianny et al., 2014) in animal bodies. On the other hand, it has also been shown that banana peels contain a variety of anti-nutritional substances, including phytates, oxalate, hydrogen cyanide, and others, which may negatively impact nutrient digestion (Sugiharto et al., 2020). Hence, despite of its high nutritional value, the use of banana plant has been restricted in broiler chicken due to its high fiber content and presence of antinutritional and toxic compounds (Sugiharto et al., 2019). Fermentation is a simple procedure that can be used to enhance the nutritional and functional characteristics of plant leaves. Food products' nutritional value and health benefits are increased by the fermentation process (Taveira et al., 2021). Several studies indicated that fermentation increased crude protein content (Koni et al., 2024) but decreased crude fibre content (Sugiharto et al., 2015, 2016), several ANF and toxic compounds in feed ingredients (Xu et al., 2012; Koni et al., 2024). In the field of pig nutrition, fermenting foods has been somewhat widespread for many years (Canibe and Jensen, 2012), However, there is a growing interest in adding fermented feed into broiler rations in order to take advantage of the good effects that it has, notably on the health of the gut and the parameters that govern output that are important (Alshelmani et al., 2016; Zhang, et al., 2016). Hence, like other high fibrous poultry feed ingredients, different parts of banana plant could be efficiently used after fermentation in commercial poultry feed due to its anti-bacterial, anti-oxidant and antibiotic activities. The fermented banana stem could enhance nutritional quality and thus improves growth performance in poultry. Inclusion of fermented banana stem powder in poultry feed might result in increased digestibility and thereby causes better overall health and productivity. Fermented feed ingredients is reported to have lower pH and higher organic acids, which might result feed from pathogen contamination prior to feeding (Niba et al., 2009), benefit chicken gastrointestinal health (Missotten et al., 2013; Sun et al., 2013) and chicken growth and development (Xie et al., 2016)).The study examined the use of banana peel waste to produce bioethanol and other high-value compounds and found that it might reduce environmental difficulties and valorize food waste (Klein et al., 2024). This study looked into whether banana peel, which is a common type of agricultural trash in Indonesia, could be used as fish food because fish need a certain amount of protein. It had a big effect on the fermentation of banana leaves, which made the protein content rise by up to 4.04 % and the fiber content fall by up to 0.69 % (Fatmawati et al., 2018). In terms of cost savings, the replacement of expensive traditional feedstuffs such as yellow maize in broiler diets may further encourage the use of cheaper unconventional fermented feedstuffs in broiler nutrition (Sugiharto et al., 2015, 2016). One another study sought to assess the nutritional value of a banana stem subjected to anaerobic fermentation and supplemented with nitrogen (N), sulfur (S), and phosphorus (P). It was observed that the supplementation of 3.00 % N, 0.40 % S, and 0.25 % P yielded the greatest protein content at 8.98 % (Rochana et al., 2017). The incorporation of fermented banana peel and banana pulp residue into the diet of domestic chicken at a rate of ten percent and twenty percent, respectively, led to an increase in the percentage of pectoral and thigh muscles as well as the total fatty acid content of chest meat (Li et al., 2024). The body weight gain and feed intake although showed a decreasing trends in crossbred native chicken fed with 10 and 20 % fermented banana peel up to 8 weeks of age, the corresponding values were comparable with control group, indicated that fermented banana peel could be fed without any significant effect in their body weight gain and feed intake in native chicken (Koni et al., 2024). Dietary inclusion of fermented banana leaves at 10 % level improved daily weight gain, feed efficiency and carcass yield in broiler chicken (Mandey et al., 2015). Further when fermented banana peel was fed to crossbred native chicken at the rate of 30 %, the body weight gain and feed consumption were significantly decreased up to 8weeks of age as compared to control group (Koni et al., 2024). The presence of antinutritional factors (tannin) at increasing levels of fermented banana peel in the diet may be the cause of the decreasing trends in body weight gain and feed intake, as tannin has astringent and bitter flavors. The incorporation of fermented banana stems at the level at 500 g/kg DM diet resulted in the higher nitrogen retention, average daily gain and lower feed conversion ratio in Kandol pigs (Huy et al., 2021). On the other hand, the dietary fermented banana stems did not exhibit any effects that were statistically significant on the hanging carcass, the dressing carcass, the pH, the color score, the marbling score of the meat, or the water holding capacity (Huy et al., 2021). The fermented banana stem while used as probiotics in fish feed (3.0 ml/100 g of feed) resulted in improved growth and survivability among carp fry (Amalia, 2023). Flow diagram depicted the process of fermented banana feed and its effects in poultry (Fig. 3).

## The utilization of banana plant extract to produce silver nanoparticles and their application in poultry feed

Parts of banana trees were successfully used to create silver and gold nanoparticles, which shown encouraging antibacterial qualities. The most researched banana by-product for these uses is banana peel (Mostafa, 2021). Banana peels, which are naturally abundant in polymers including lignin, hemicellulose, and pectins, have the potential to be utilized in the production of silver nanoparticles (Emaga et al., 2008). The extract from banana peels and AgNO3, silver nitrate solution. These AgNPs produced notable effects against several strains of bacterial pathogens (Iqbal et al., 2023).

AgNPs, or silver nanoparticles, are important materials that have drawn a lot of interest. These nanoparticles are employed in drug administration, biological imaging, medical engineering, and catalysis due to their special optical, thermal, electrical, and biological characteristics, which raises their demand (Jain et al., 2008). Several research groups have documented creating AgNPs with plant extracts acting as capping and lowering agents (Shankar et al., 2004; Vidhu and Philip, 2015). Gram-positive bacteria (*Lactobacillus* species, *Leuconostoc lactis, Actinomyces naeslundii*) were considerably more prevalent in quail birds that were given water with 25 mg/kg Ag-nano (Sawosz et al., 2009). Traditionally, silver compounds have been used to stop bacterial growth (Wadhera and Fung, 2005).

Many polymers found in banana peels, including cellulose, hemicellulose, lignin, and pectin, may be used to create silver nanoparticles (Emaga et al., 2007). Microwave-assisted biosynthesis can quickly and easily produce AgNPs using plant extracts as reducing and stabilizing agents. Microwave irradiation quickly warms the reaction media, creating monodispersed nanoparticles (Nadagouda et al., 2011). In nanotechnology, banana peel has been used to prepare silver nanoparticles and as a source of cellulose nanofibers (Tibolla et al., 2018). Green nanoparticle synthesis is a potential approach of recycling banana by-products by using them as capping and reducing agents in the nanoparticle production process rather than using conventional chemical procedures (Ibrahim, 2015). When compared to the aqueous peel extract, (Kokila et al., 2015) found that the biosynthesized silver nanoparticles from the BPE of Cavendish bananas exhibited high DPPH radical and ABTS scavenger activity. The well diffusion test indicated that the antibacterial activity of these nanoparticles against *Klebsiella pneumoniae, E. coli*, and *S. aureus* was observed in ascending order of inhibition area. Silver nanoparticles made from banana stem waste were demonstrated to have strong antibacterial properties against *E. coli* by (Dang et al., 2017) with an inhibition area of 12 mm. In the meantime, titanium nanoparticles were made by (Hameed et al., 2019) using banana peel extract (at 50 %). Using the well diffusion approach, the collected particles demonstrated antimicrobial activity against a range of pathogens, with *S. aureus* and *E. coli* showing the largest inhibition zones (Mahmoud, 2012).

Silver nanoparticles (nano-Ag) represent a promising alternative feed additive for poultry and potentially for medical uses. Nano-silver may influence the metabolic activity and health of animals. Multiple assumptions underpin the application of silver nanoparticles in the poultry business.

1. Nanoparticles of silver can boost cell immunity by promoting heat shock protein (HSP) synthesis without activating pro-inflammatory pathways.
2. Nano-Ag would enhance oxygen demand and elevate metabolic rates, hence facilitating the growth and development of embryos.
3. Nano-Ag can influence the gene expression of Fibroblast Growth Factor (FGF), which promotes the proliferation and differentiation of blood vessels, muscle, and fibroblast cells.
4. Nano-Ag has antibacterial capabilities that may influence microbial populations without causing resistance and may boost anabolic activity, promoting animal development and growth.

**Fig. 4:**
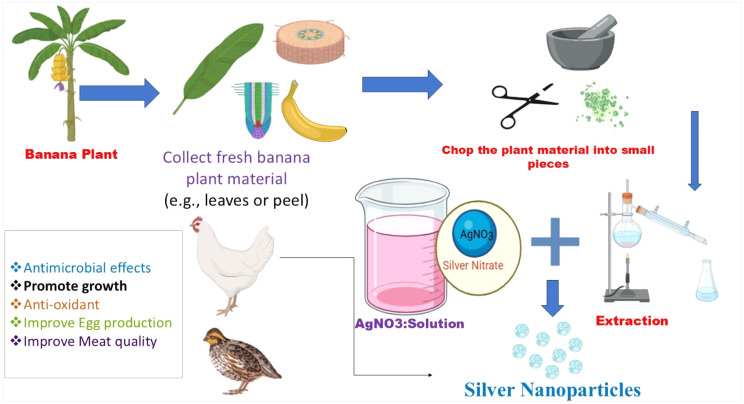
Flow diagram emphasizing the synthesis of silver nanoparticles using banana plant extract and their application in poultry.

It is advised to utilize nanosilver as anti-microbial agent in the poultry sector under great care. Silver nanoparticles (nano-Ag) produced by the banana plant can be used as an alternate growth-promoting supplement for poultry production. Flow diagram emphasizing the synthesis of silver nanoparticles using banana plant extract and their application in poultry (Fig. 4). Thus, it requires further work ahead in poultry industry.

Future Perspectives

Though their potential is great, numerous issues have to be resolved to utilize the advantages of fermented banana plants in poultry diet:

¨ Consistent feed quality depends on optimizing fermentation conditions including microbial strains, time, and substrate preparation.
¨ Although banana plants offer great nutrients, their composition in poultry diets has to be properly combined with other vital feed components to satisfy poultry nutritional needs.
¨ Large-scale manufacturing and incorporation into commercial chicken feed call for more feasibility research and financial evaluations.
¨ Formulating safety protocols and regulatory structures for the integration of fermented banana plants into poultry feed is crucial for industry acceptance.

## Conclusion

The banana contains significant amounts of fiber, proteins, potassium, cellulose, hemicelluloses, lignin, starch, resistant starch, polyunsaturated fatty acids, and essential amino acids. It has a lot of sugar and is often used to feed animals. Banana peel has 10.09 % protein, 18.01 % crude fiber, 5.17 % fat, 55.59 % dry matter, 0.36 % calcium, 0.10 % phosphorus, and 3727 kcal/kg gross energy. There are B6, C, and E vitamins in banana peel as well. Under heat stress, vitamin C can function as an antioxidant, while serotonin is believed to have an antidepressant role, leading to an increase in feed intake and body weight. The weight of the 35-day-old spleen increased when fed with banana peel meal. Because banana peels being considered are a valuable source of prebiotics, antioxidants, and pro-vitamin A. Additionally, the extract from banana peels shown antibacterial action against *Staphylococcus aureus, Aggregatibacter actinomycetemcomitans*, and *Porphyromonas gingivalis*. By using fermented banana plant preservation as poultry feed (Better efficiency in turning feed into energy; Better growth results; Probiotics aid intestinal health; cost-effective alternative feed source; sustainability and excellent for the environment). Adding 10 % banana peel to poultry feed improved feed efficiency, conversion, and egg and meat quality. Therefore, these characteristics of banana peel may be advantageous for poultry and livestock’s health and welfare. This review will inspire poultry nutritionists because few studies have examined the effects of adding fermented whole banana plant and its residue to poultry diets, especially on feed quality and taste, making it a promising alternative feedstuff.

## Disclosures

The authors say they don’t have any known personal or financial relationships or financial interests that could have seemed to affect the work in this study.

## Declaration of competing interest

The authors declare that they have no conflicts of interests.
